# Impact of intense sanitization on environmental biofilm communities and the survival of *Salmonella enterica* at a beef processing plant

**DOI:** 10.3389/fmicb.2024.1338600

**Published:** 2024-02-16

**Authors:** Rong Wang, Manita Guragain, Sapna Chitlapilly Dass, Vignesh Palanisamy, Joseph M. Bosilevac

**Affiliations:** ^1^U.S. Department of Agriculture, Agricultural Research Service, Roman L. Hruska U.S. Meat Animal Research Center, Clay Center, NE, United States; ^2^U.S. Department of Agriculture, Agricultural Research Service, Eastern Regional Research Center, Wyndmoor, PA, United States; ^3^Department of Animal Science, Texas A&M University, College Station, TX, United States

**Keywords:** multispecies biofilms, sanitization, *Salmonella enterica*, 16S rRNA amplicon gene sequencing, environmental bacteria, meat safety

## Abstract

*Salmonella enterica* is a leading cause of foodborne illness in the U.S. In the meat industry, one action taken to address pathogen contamination incidence is an intense sanitization (IS) of the entire processing plant that many large processors perform annually or semiannually. However, this procedure’s immediate and long-term impact on environment microbial community and pathogen colonization are unknown. Here we investigated the impact of IS procedure on environmental biofilms and the subsequent *S. enterica* colonization and stress tolerance. Environmental samples were collected from floor drains at various areas 1 week before, 1 week, and 4 weeks after the IS procedure at a beef plant with sporadic *S. enterica* prevalence. Biofilm formation by microorganisms in the drain samples without *S. enterica* presence was tested under processing temperature. The ability of the biofilms to recruit and/or protect a co-inoculated *S. enterica* strain from quaternary ammonium compound (QAC) treatment was determined. The community structure of each drain sample was elucidated through 16S rRNA amplicon community sequencing. Post-IS samples collected from 8 drains formed significantly stronger biofilms than the respective pre-IS samples. *S. enterica* colonization was not different between the pre- and post-IS biofilms at all drain locations. *S. enterica* survival in QAC-treated pre- and post-IS mixed biofilms varied depending upon the drain location but a higher survival was associated with a stronger biofilm matrix. The 16S rRNA amplicon gene community sequencing results exhibited a decrease in community diversity 1 week after IS treatment but followed by a significant increase 4 weeks after the treatment. The IS procedure also significantly altered the community composition and the higher presence of certain species in the post-IS community may be associated with the stronger mixed biofilm formation and *Salmonella* tolerance. Our study suggested that the IS procedure might disrupt the existing environmental microbial community and alter the natural population composition, which might lead to unintended consequences as a result of a lack of competition within the multispecies mixture. The survival and recruitment of species with high colonizing capability to the post-IS community may play crucial roles in shaping the ensuing ecological dynamics.

## Introduction

In the meat industry, product contamination by foodborne pathogens such as *Escherichia coli* (*E. coli*) O157:H7 and *Salmonella enterica* (*S. enterica*) is a serious public health concern. Even though animal hides have been deemed as the main contamination source at processing plants (Barkocy-Gallagher et al., 2003; Nou et al., 2003; Small et al., 2005; Arthur et al., 2007, 2008), available results have indicated that bacterial biofilm formation at commercial plants may play an important role in some contamination events (Wang et al., 2014, 2017; Yang et al., 2015; Visvalingam et al., 2016). Furthermore, the critical impact of environmental microorganisms on pathogen tolerance and survival via mixed biofilm formation has been demonstrated. For instance, our previous study (Chitlapilly Dass et al., 2020) showed that *E. coli* O157:H7 cells that formed mixed biofilms with the environmental microorganisms from a meat plant with a high *E. coli* O157:H7 prevalence history displayed significantly stronger sanitizer tolerance. Thus, the unique composition of the environmental microflora and the synergistic and/or antagonistic interactions within the multispecies biofilms can either promote or inhibit the growth and colonization of the specific pathogens that may become the source of the contamination events.

Once contamination events take place or pathogen prevalence is identified in products or the environment, complete cleaning of the environment to eliminate all existing pathogens may be the most intuitive strategy. One of the actions taken to address contamination events of *E. coli* O157:H7 or *S. enterica* at commercial plants is an intense sanitization (IS) procedure for the entire processing environment. The IS procedure is also a part of routine food safety systems that many large processors perform annually or semiannually, which involves cleaning the entire facility including ceiling, floor, wall, sink, pipe, contact surfaces, equipment surface, and all other hard-to-reach spots/corners. However, anecdotal evidence provides mixed results on the efficacy of the IS procedure to resolve contamination events. In some cases, the problem was described as resolved, while in others, events continued or increased for a period following the treatment.

Numerous human medical facility reports described the impacts of sanitation on pathogenic and natural biofilms. Biofilms present in hospital sinks and dishwashers were found to harbor antibiotic-resistant and pathogenic bacteria that can be a source of the spread of these infections (McBain et al., 2003a,b; Raghupathi et al., 2018). However, it was reported that intervention procedures such as replacement of contaminated plumbing unexpectedly led to an increase of antimicrobial-resistant species that were targeted for elimination, likely due to the disruption of pre-existing natural biofilms that resulted in a lack of competition within the multispecies community that favored pathogen invasion (Kotay et al., 2020). Similarly, enhanced confinement and cleaning of the microorganisms on surfaces in clinical settings were found to be associated with a decrease in microbial diversity but correlated to an increase in resistance (Mahnert et al., 2019). Furthermore, rapid recolonization of antimicrobial-resistant pathogens after plumbing replacement and other intervention strategies (Tofteland et al., 2013; Aspelund et al., 2016; De Deborah et al., 2017) was reported to be associated with a wide microbial population shift, a potential result of microbial community disruption that altered the natural population composition and recruited undesirable species with high colonizing and survival capability. These observations may also be attributed to the phenomena of pulse dynamics and disturbance in microbial ecology (Jentsch and White, 2019), that a sudden disturbance to the ecological niche might lead to certain temporary changes, such as increased contamination events or antimicrobial resistance dissemination, as the result of community structure shift followed by the recovery period when the disturbance activity is resolved.

The above studies suggest that environmental biofilms can harbor pathogens and that disrupting such a natural community may have unintended effects. However, the literature is limited with regard to the effects of sanitizing strategies on naturally occurring environmental biofilms and the subsequent impact on pathogen prevalence at meat processing plants or in the health care system. Therefore, the present study aims to directly examine the effects of the IS treatment on processing plant environmental biofilms before and after such procedure, and further follow the treated locations over time to monitor how the biofilms recover and interact with pathogens (*S. enterica*).

## Materials and methods

### Intense sanitization of the beef plant

A beef processing plant with sporadic *Salmonella* incidence was selected for this study. The IS procedure was performed by the plant sanitation and maintenance personnel using the multicomponent sanitizer Decon7 (Decon7 Systems Inc. Coppell, TX) following the recommendations and guidelines provided by the Decon7 manufacturer, which was proceeded by a thorough cleaning of the processing environment and removal of all visible soil and built-up materials. Application of Decon7 was through a generated foam left in contact with surfaces for 8 h. The Decon7 treatment was followed by a rinse using 120–125°C water, and a final antimicrobial treatment of 200 ppm peroxyacetic acid (Blitz; Evonic Corp. Parsippany NJ).

### Floor drain sample collection and characterization

Floor drain samples were collected 1 week before, 1 week, and 4 weeks after the IS procedure using cellulose sponges (Speci-sponge; Nasco, Atkinson WI) each wetted with 10 mL of buffered peptone water (BPW). Drains were standard industrial drains located at low points of the floor for the collection and removal of runoff, or trench drains which are a trough spanning the floor that carries runoff to a central basin that enters plumbing (Table 1). The selected drains on opposite sides of the hotbox, cooler, grinder room, and further processing areas were at least 25 m apart and did not share drainage lines. After an appropriate drain was identified, its covering grate was removed and an area of ~500 cm^2^ was vigorously swabbed with the sponge, turning it over halfway through the process. The underside of the grate and interior surfaces were sampled to collect bacteria and biofilms. Sponges were sealed in their whirl-pak bag and then transported to the laboratory on wet ice in a cooler. To ensure an adequate sample was obtained, each drain sample was thoroughly hand massaged then portions were removed and serially diluted to measure the levels of total mesophile count (TMC), and psychrophilic bacteria (PB), as well as Enterobacteriaceae (EB), coliforms (*CF*) and *E. coli* (EC) using Petrifilm (3 M, St Paul, MN). Petrifilm aerobic count (AC) plates were used for TMC by incubating 48 h at 30°C, and PB by incubating 10 days at 5°C; Pertrifilm EB was used for EB, while Petrifilm EC was used for *CF* and EC according to the package insert.

**Table 1:** Floor drain samples collected from a beef processing plant.

Sample	Location	Drain type
1	Grinder room	Trench drain
2	Grinder room	Trench drain
3	Combo bin storage	Trench drain
4	Fabrication floor	Floor drain
5	Fabrication floor	Trench drain
6	Sales cooler	Floor drain
7	Sales cooler	Trench drain
8	Fabrication cooler	Trench drain
9	Hot box	Trench drain
10	Hot box	Trench drain
11	Hot beef hall	Floor drain
12	Hot Scale	Floor drain

### Culture conditions for drain samples and the *Salmonella enterica* strain

One *S. enterica* Montevideo strain (MARC-5B) previously isolated from the beef product at this processing plant was stored at −70°C in Lennox Broth (LB, Acumedia Manufacturers, Baltimore, MD) without salt (LB-NS) medium containing 15% glycerol. For each experiment, the *Salmonella* strain was streaked from the glycerol stock onto Tryptic Soy Agar (TSA) (Difco, Beckton Dickinson, Sparks, MD) plates and grown overnight at 37°C, then one single colony on the plate was inoculated into LB-NS medium and grown overnight at 37°C with orbital shaking at 200 rpm to reach bacterial stationary phase containing a cell concentration of approximately 5 × 10^8^ cells/mL. The bacterial broth culture was then further diluted in fresh sterile LB-NS medium for each experiment.

Thirty-six drain samples were collected 1 week before, 1 week, and 4 weeks after the IS procedure at twelve floor drains starting from the “clean” or chilled side of the plant (processed beef products) and proceeding toward the harvest floor portion of the plant. All samples were confirmed to be free of *S. enterica* presence (Table 1). To best maintain the original microbial composition of the floor drain samples and expand the sample volume for experimental use, each sample was diluted 1:50 in LB-NS medium and incubated at 7°C (similar to chilled processing plant environmental temperature) for 5 days with orbital shaking at 200 rpm, then aliquoted and stored at −20°C in LB-NS medium with the addition of sterile glycerol to 15%.

### Biofilm formation by floor drain samples on 96-well polystyrene microtiter plates

The biofilm-forming ability of the floor drain samples was measured on 96-well polystyrene plates with Crystal Violet (CV) staining as previously described (Wang et al., 2014) with modifications. Briefly, the glycerol stocks of the floor drain samples were thawed and inoculated 1:1000 into sterile LB-NS medium then incubated at 7°C for 5 days with orbital shaking at 200 rpm. The 5-day cultures were 100-fold diluted in sterile LB-NS medium and added to 96-well flat-bottom polystyrene plates (Costar, Corning, NY) at 200 μL per well. Control wells contained only sterile LB-NS medium. Plates were incubated statically for another 5 days at 7°C. After incubation, supernatants were gently removed by aspiration and the plates were washed with 200 μL per well of sterile water to remove loosely attached cells, air dried for 5 min, then stained with 200 μL per well of 0.1% CV for 20 min at 22 to 25°C. The plates were washed again and air dried as described above, then the remaining CV was dissolved in 100 μL per well of 85% ethanol. The amount of the extracted CV in each well was determined by measuring A_570_ using a microplate reader (Molecular Devices, Sunnyvale, CA). Each data point was averaged from at least five replicate wells. The experiments were performed three times using independent cultures.

### *Salmonella enterica* colonization and post-sanitization survival in multispecies biofilms

To measure *Salmonella* colonization within the multispecies biofilms and to investigate the potential impact of the interspecies interactions between the *Salmonella* cells and the environmental microorganisms on sanitizer tolerance of the *S. enterica* strain in mixed biofilms, a quantitative biofilm assay using colony enumeration was performed using 96-well polystyrene plates as previously described (Wang et al., 2013) with modifications. Briefly, the *S. enterica* Montevideo strain isolated from the experimental processing plant was grown overnight at 37°C in LB-NS medium to reach a cell concentration of approximately 5 × 10^8^ CFU/mL then diluted 1:100 into the floor drain samples that had been cultured for 5 days at 7°C as described above. The diluted drain cultures containing the *Salmonella* strain were added to the 96-well polystyrene plates at 200 μL per well and incubated statically for another 5 days at 7°C. At the end of the incubation period, supernatants were gently removed by aspiration, and the plates were washed with 200 μL of sterile water per well to remove any loosely attached cells. The plates were then filled with 200 μL/well of a commercial quaternary ammonium chloride–based sanitizer Vanquish ™ (Dawn Chemical Corp., Milwaukee, WI) that was prepared at 300 ppm (recommended concentration) and incubated for 1 min (recommended minimal surface exposure time) in order to test and compare *Salmonella* stress tolerance in the pre- and post-IS mixed biofilms. Positive control samples were treated with 200 μL/well of sterile water.

After the 1 min incubation, the supernatants were gently removed, and each well was washed with 200 μL sterile water. To neutralize the sanitizer activity, the plates were filled with 200 μL/well of sterile Dey/Engley (D/E) broth (BBL, Difco, Sparks, MD) supplemented with 0.3% soytone and 0.25% sodium chloride. The biofilm cells were harvested by scraping the surface of the well with sterile pipette tips and rinsing the wells with the D/E broth. Cells harvested from replicated wells were combined and vigorously vortexed to disrupt cell aggregates, then serially diluted in fresh D/E broth and plated onto xylose lysine deoxycholate (XLD; Oxoid Ltd., Hampshire, England) agar plates for colony enumeration after overnight incubation (16 to 18 h) at 37°C. *Salmonella* cells in the multispecies biofilms were distinguished from background microorganisms by colony morphology on XLD plates (black colonies) so that the amount of *Salmonella* cells in mixed biofilms was calculated using CFU counts of the black colonies on the plates and the corresponding dilution factors. The experiments were repeated three times using independent cultures, and each sample was tested in five replicates in each experiment.

### DNA extraction and 16S rRNA gene amplicon-based community sequencing

The floor drain samples were enriched in LB-NS medium for 5 days at 7°C as described above, then DNA extraction/purification was performed for amplicon sequencing based on the variable region V4 of the 16SrRNA gene as previously described (Chitlapilly Dass et al., 2020). Briefly, primers used were 15F (5’-GTGCCAGCMGCCGCGGTAA-3′) and 806R (5’-GGACTACHVGGGTWTCTAAT3’), flanking the 515 and 806 regions. Barcodes were attached to the 806R primers. Library preparation and 2 × 250 bp paired-end sequencing were carried out using the Illumina® MiSeq® platform at Novogene (Sacramento, CA) as described previously (Chitlapilly Dass et al., 2020).

### Statistical and bioinformatics analysis

Total mixed biofilm matrix measured as O.D _570 nm_ and the logarithmic cell counts (log_10_ CFU/mL) of *S. enterica* in mixed biofilms were analyzed and compared using a one-way analysis of variance (ANOVA) with a post-Dunnett’s multiple comparisons test using GraphPad Prism software (GraphPad Software, La Jolla, CA). *p* values lower than 0.05 were considered statistically significant.

Analysis of 16S rRNA amplicon gene community sequencing results was conducted using Quantitative Insights Into Microbial Ecology (QIIME2.0). Paired-end sequences were de-multiplexed using MiSeq Control software prior to importing into QIIME. The sequences were classified using Silva_138 release as the reference database with the pre-trained classifier based on 99% sequence identity.^1^ The downstream analyses were performed on the final sequence count and taxonomy table in RStudio using different packages of R *viz.* Percent relative abundance of each genus in each sample was tabulated with Microsoft Excel. Linear Discriminant analysis (LDA) Effect Size (LEfSe) analysis was carried out using the web platform MicrobiomeAnalyst^2^ with a log LDA score cutoff of less than 2.0 and *p* < 0.05.

Qiime2 environment’s ‘mafft’ and ‘FastTree’ were used to construct phylogenetic tree and generate tree file. The alpha and beta diversity of the datasets were then calculated based on different metrics such as Chao1, Shannon, weighted and unweighted Unifrac distances. A Kruskal-Wallis test was performed to test the significance of the alpha diversity results. Similarly, a Permutational multivariate ANOVA (PERMANOVA) was performed for beta diversity analysis.

## Results

### Biofilm development by floor drain samples

**Figure 1:**
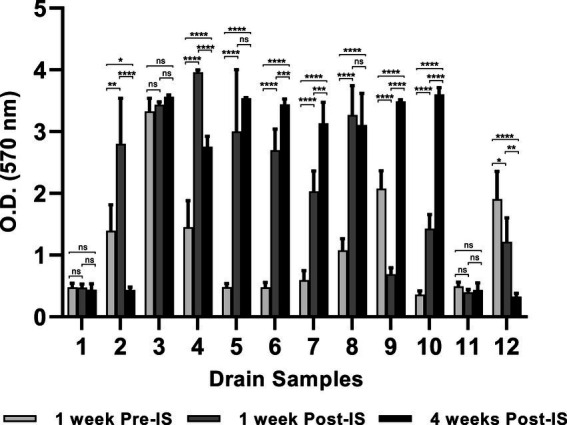
Mixed species biofilms developed on 96-well polystyrene plate. Floor drain samples were enriched in LB-NS medium for 5 days at 7°C, then allowed to form biofilms on 96-well polystyrene plates in LB-NS medium at 7°C for 5 days. Data are shown as mean O.D _570 nm_ ± standard deviation, *n* = 5. Statistical analysis is performed using a one-way analysis of variance (ANOVA) with a post-Dunnett’s multiple comparisons test. ns: no significance; *p < 0.05; **p < 0.01; ***p < 0.001; ****p < 0.0001.

The environmental microorganisms in most of the drain samples exhibited positive biofilm-forming ability which varied depending upon the drain locations and the time points when the samples were collected (Figure 1). Of the twelve pre-IS samples (Trip 1), six samples (Sample #1, #5, #6, #7, #10, and #11) exhibited weak biofilm formation (O.D _570 nm_ ≤ 0.5), meanwhile, samples #3, #9, and #12 developed strong biofilms (O.D _570 nm_ ≥ 2.0) with sample #3 from combo bin storage being the strongest biofilm former (O.D _570 nm_ = 3.3).

Biofilm formation by the three samples collected from the same drain 1 week before, 1 week, and 4 weeks after IS were compared. The microorganisms collected from drains in the grinder room (#1) and hot beef hall (#11) all exhibited weak biofilm-forming ability and did not differ across the three sampling time points. However, the IS procedure showed a profound impact on biofilm formation by microorganisms harvested from other locations. Most samples collected 1-week post-IS showed significantly enhanced biofilm formation compared to their pre-IS counterparts except hotbox drain #9 and hot scale drain #12 which both exhibited decreased biofilm mass 1 week after IS and the combo bin storage drain #3 which showed no significant difference in biofilms formed by samples collected before and 1 week after IS (Figure 1).

Samples collected from cooler drains #6 and #7, and hotbox drains #9 and #10 at 4 weeks after IS all formed significantly stronger biofilms than the pre- and 1-week post-IS samples harvested from the same drains. Conversely, microorganisms collected 4 weeks post-IS from drains #2 (grinder room) and #12 (hot scale) showed significantly reduced biofilm-forming ability compared to the samples collected from each of these 2 drains pre- and 1-week post-IS. Samples from fabrication floor drain #5 and the nearby cooler drain #8 at 4 weeks post-IS showed significantly higher biofilm formation compared to the same drain pre-IS samples but not different from their respective 1-week post-IS samples. No significant difference among the biofilms formed by the three samples from combo bin storage drain #3 which all appeared to be strong biofilm formers. The sample from fabrication floor drain #4 at 4 weeks post-IS exhibited lower biofilm-forming ability than the 1-week post-IS sample but was still significantly stronger than the sample collected from the same drain before the IS procedure.

### *Salmonella enterica* colonization and survival within drain mixed biofilms

The inoculated *S. enterica* strain was able to efficiently colonize within the mixed biofilms that contained *Salmonella* cells ranging from 2.8–4.6 log_10_ CFU cells (Table 2). Despite the variation in biofilm-forming ability among samples collected from the 3 sampling time points, *Salmonella* colonization was not different in the mixed biofilms formed by samples collected from the same drain location before, 1 week, and 4 weeks after the IS procedure. The only significant difference in *S. enterica* colonization among the three mixed biofilm communities was observed in hot scale drain #12 (Table 2). *Salmonella* colonization in the mixed biofilm formed by the 4-week post-IS sample from drain #12 was significantly higher than that in the mixed biofilm by its pre-IS sample.

**Table 2:** Biofilm formation by floor drain samples and *S. enterica* cell density in multispecies drain biofilms after sterile water or 300 ppm QAC treatment.

Sample	Biofilm formation			*S.enterica*: water treated			*S. enterica*: QAC treated		
	Pre-IS	1w post-IS	4w post-IS	Pre-IS	1w post-IS	4w post-IS	Pre-IS	1w post-IS	4w post-IS
1	0.5 (0.1)	0.5 (0.1)	0.4 (0.1)	4.1 (0.4)	4.3 (0.6)	4.1 (0.5)	ND	ND	0.3 (0.6)
2	1.4 (0.4)	2.8 (0.7)	0.4 (0.04)	4.4 (0.5)	3.0 (1.4)	4.1 (0.8)	0.9 (0.9)	ND	ND
3	3.3 (0.2)	3.4 (0.04)	3.6 (0.02)	3.8 (0.3)	4.0 (0.8)	3.5 (0.2)	0.3 (0.6)	0.3 (0.6)	1.4 (0.3)
4	1.5 (0.4)	4.0 (0.04)	2.8 (0.2)	4.1 (0.8)	4.6 (0.3)	4.2 (0.9)	0.3 (0.6)	2.8 (1.3)	0.9 (0.9)
5	0.5 (0.1)	3.0 (0.9)	3.5 (0.01)	3.7 (0.3)	3.3 (0.5)	3.2 (0.6)	0.5 (1.0)	ND	ND
6	0.5 (0.1)	2.7 (0.3)	3.4 (0.1)	3.3 (0.3)	3.6 (0.8)	3.9 (0.5)	1.2 (1.1)	0.9 (0.8)	0.3 (0.6)
7	0.6 (0.2)	2.0 (0.3)	3.1 (0.3)	3.4 (0.6)	3.6 (0.4)	3.0 (0.8)	1.2 (0.2)	1.0 (0.9)	1.3 (0.3)
8	1.1 (0.2)	3.3 (0.5)	3.1 (0.5)	3.7 (0.4)	3.3 (0.2)	3.9 (0.6)	1.0 (1.1)	0.8 (0.7)	2.1 (0.2)
9	2.1 (0.3)	0.7 (0.1)	3.5 (0.02)	3.2 (0.4)	3.1 (0.4)	3.2 (0.1)	1.3 (0.3)^c^	ND^d^	1.6 (0.7)^c^
10	0.4 (0.1)	1.4 (0.2)	3.6 (0.1)	3.0 (0.6)	2.8 (0.8)	3.3 (0.2)	0.3 (0.6)	0.8 (0.7)	1.8 (0.5)
11	0.5 (0.1)	0.4 (0.05)	0.4 (0.1)	3.3 (0.5)	3.7 (0.2)	3.5 (0.4)	ND	0.3 (0.6)	0.4 (0.8)
12	1.9 (0.4)	1.2 (0.4)	0.33 (0.1)	3.1 (0.2)^a^	3.6 (0.5)^ab^	4.0 (0.3)^b^	0.3 (0.6)	0.3 (0.6)	ND

Data of biofilm formation are shown as mean O.D _570 nm_ (*n* = 5). Data of *S. enterica* cell density are shown as mean log_10_ CFU/mL (standard deviation) (*n* = 3). Mixed biofilm formation and sanitizer tolerance assays were performed on 96-well polystyrene plates as described in Materials and Methods. ND: not detected, indicating that viable Salmonella cells were below the limit of detection (1.0 log_10_ CFU/sample). Data were analyzed using a one-way analysis of variance (ANOVA) with a post-Dunnett’s multiple comparisons test. Statistical analysis of biofilm formation data is shown in Figure 1. Values labeled with different letters in the same row of each sub-table of *S. enterica* cell density are statistically different (*p* < 0.05).

*S. enterica* survival after quaternary ammonium compound treatment in the multispecies biofilms varied with drain locations and the time points when the samples were collected. Within each set of the three samples collected from the same drains, a trend of positive association between high *Salmonella* survival and strong biofilm matrix was observed in samples from drains #2, #4, #8, #9, #10, and #12 even though the difference in *Salmonella* survival within each set of the three mixed biofilm communities did not reach statistical significance except for the three samples collected from hotbox drain #9. Statistical significance was detected in the results of both *Salmonella* survival and biofilm matrix among these 3 samples. However, such a positive association between mixed biofilm formation and *Salmonella* survival was not observed in the 3-sample sets collected from fabrication floor drain #5 and cooler drains #6 and #7 (Table 2; Figure 1).

### 16S rRNA amplicon gene analysis of the mixed biofilm communities

The thirty-six drain samples collected at the twelve different locations from the 3 sampling trips were enriched and analyzed to assess the overall bacterial community structure differences before and after the IS procedure. Microbial taxa were compared based on the three sample collection time points and the six areas of the drain locations.

**Figure 2:**
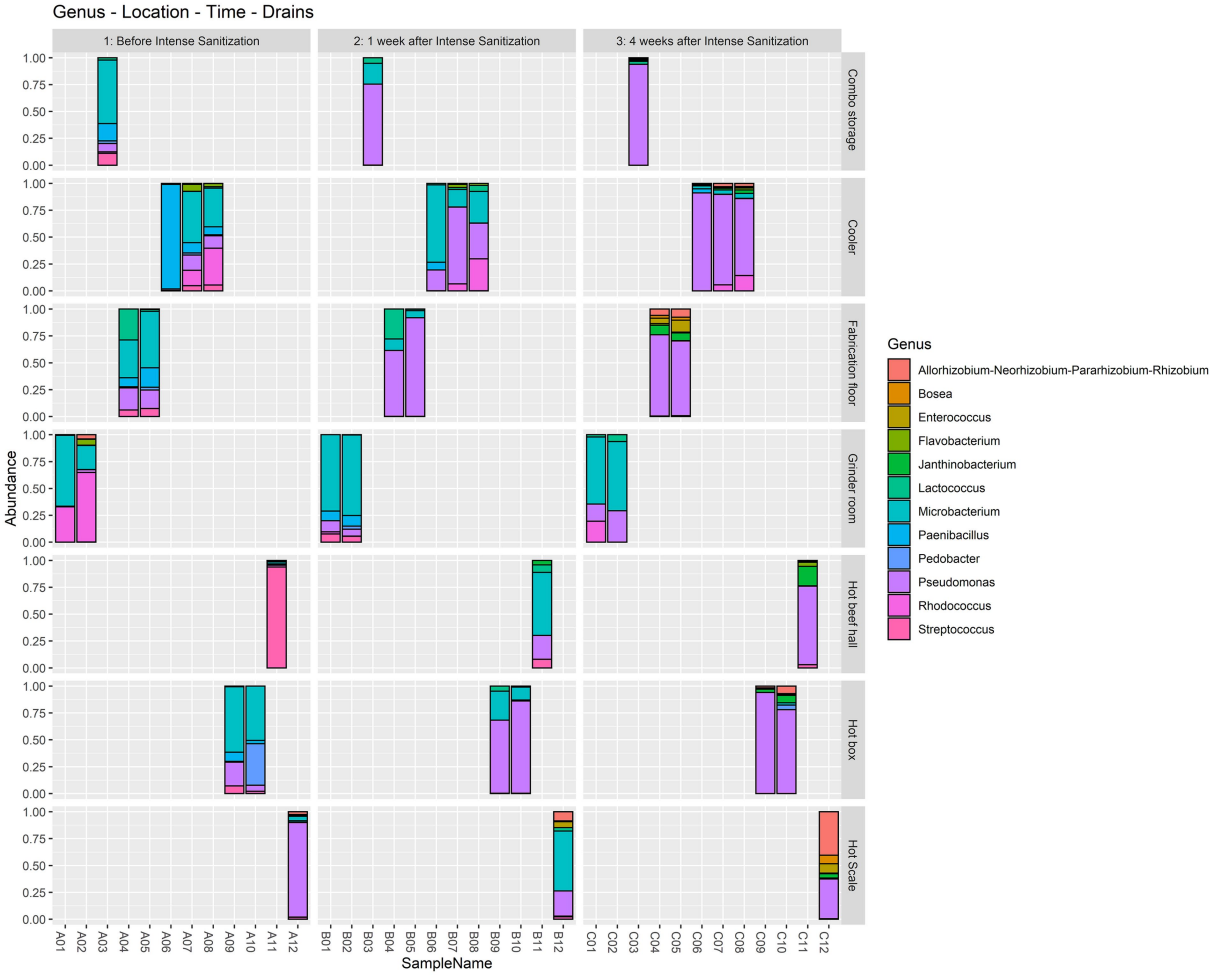
Relative abundance at the genus level in the floor drain samples. The floor drain samples were enriched in LB-NS medium at 7°C for 5 days and analyzed with 16S rRNA gene amplicon sequencing to demonstrate the relative abundance of the microorganisms in the samples before, one week, and four weeks after the IS procedure.

At the genus level, *Microbacterium* was highly dominant (> 45%) in the combo bin storage sample before IS but was overtaken by *Pseudomonas* 1 and 4 weeks after sanitization. One pre-IS cooler drain sample had a dominance of *Paenibacillus* (> 97%) which was replaced by *Microbacterium* 1 week after IS and by *Pseudomonas* 4 weeks after IS. The other two pre-IS cooler drains had *Microbacterium* (> 35%) and *Rhodococcus* (> 33%) which were overtaken by *Pseudomonas* 1 and 4 weeks after IS. Both pre-IS fabrication floor drain samples were dominated by *Microbacterium* (> 34%) and one sample had a considerable abundance of *Lactococcus* (28%). However, they were surpassed in abundance by *Pseudomonas* 1 and 4 weeks after IS. Both grinder room samples had a high abundance of *Rhodococcus* and *Microbacterium* before IS followed by a further increase of *Microbacterium* 1 and 4 weeks after sanitization. The pre-IS hot beef hall sample had a dominance of *Streptococcus* which was replaced by *Microbacterium* 1 week post-IS and *Pseudomonas* 4 weeks post-IS. Both pre-IS hotbox samples had a high abundance of *Microbacterium* and *Pedobacter* in one of the hotbox drains, which was overtaken by *Pseudomonas* 1 and 4 weeks after IS. The pre-IS hot scale sample had a high dominance of *Pseudomonas* which was shared by *Microbacterium* 1 week post-IS followed by a *Rhizobium* abundance increase 4 weeks after IS (Figure 2).

**Figure 3:**
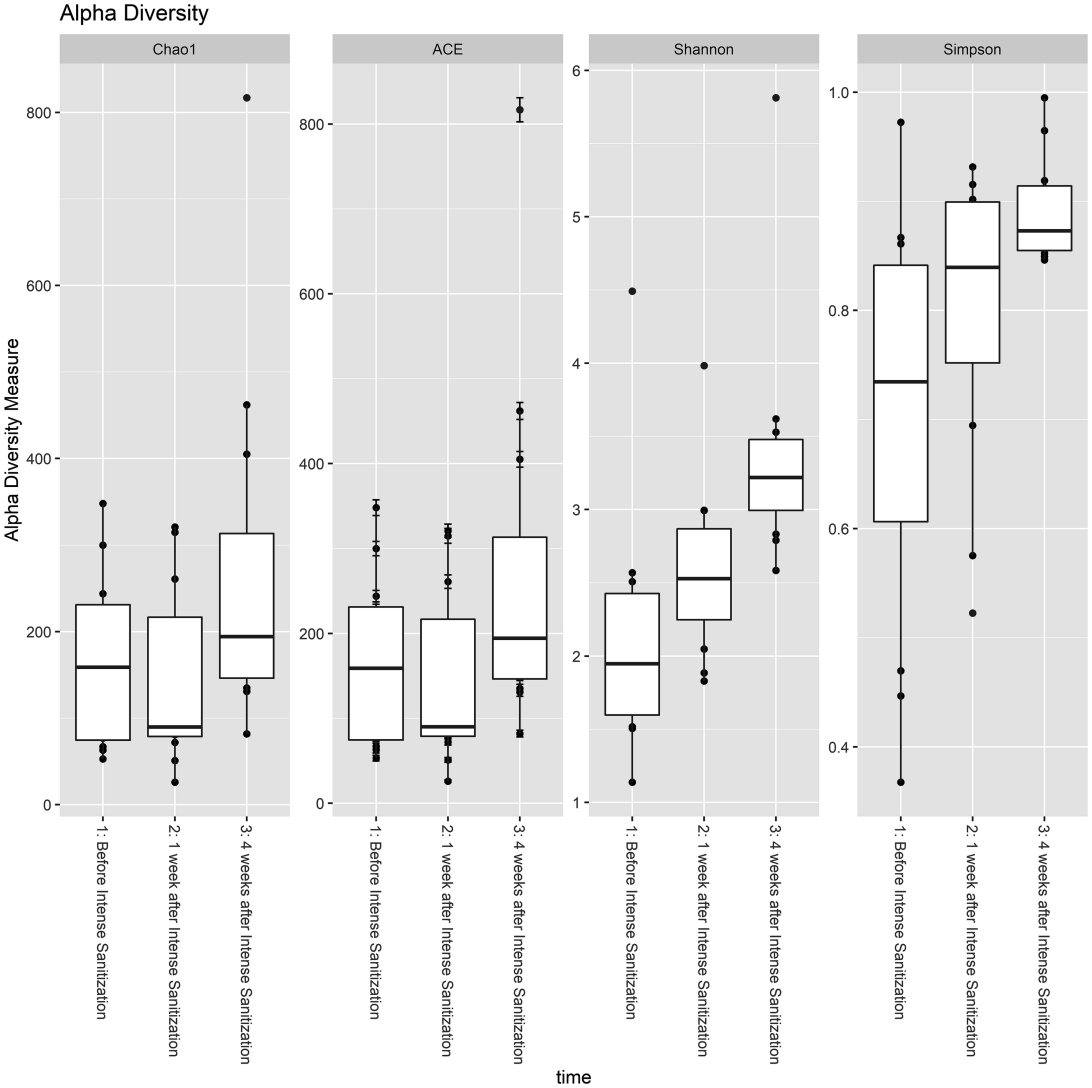
Alpha diversity analysis of floor drain samples collected before, 1 week, and 4 weeks after IS procedure. The boxplot represents the interquartile range of the alpha diversity measures with the dashed line inside the box representing the median value of alpha diversity. Each boxplot represents the central tendency and the spread of the alpha diversity across different times of sanitization.

Based on four diversity metrics including Shannon, Simpson, ACE, and Chao1 indexes, diversity analysis revealed that drain samples collected 1 week after the IS procedure had the least alpha diversity. However, samples taken 4 weeks after the procedure had significantly increased, and therefore the highest alpha diversity as the Kruskal-Wallis test based on the Shannon index yielded statistically significant (*p* < 0.05) results in the variation of alpha diversity among the samples collected at the three time points (Figure 3; Table 3).

**Table 3:** Variation of alpha diversity between time after IS procedure analyzed with Kruskal-Wallis test based on the Shannon index.

Group 1	Group 2	H	*p*-value	*q*-value
1 week after IS (*n* = 12)	4 weeks after IS (*n* = 12)	8.333333	0.003892	0.005839
1 week after IS (*n* = 12)	Before IS (*n* = 12)	4.32	0.037667	0.037667
4 weeks after IS (*n* = 12)	Before IS (*n* = 12)	12.40333	0.000429	0.001286

Meanwhile, the PERMANOVA analysis investigating beta diversity for both unweighted and weighted unifrac metrics showed a significant statistical difference (*p* < 0.05) among the three groups (Table 4).

**Table 4:** PERMANOVA analysis for beta diversity based on Unweighted and Weighted unifrac metrics.

	Unweighted unifrac	Weighted unifrac
Sample size	36	36
Number of groups	3	3
Test statistic	2.51815	3.26759
*P* value	0.001	0.006
Number of permutations	999	999

## Discussion

Intense sanitization of the entire plant has been implemented by major meat processors as an intervention strategy in the hope of eliminating any existing colonized pathogens and reducing the overall environmental microbial load to a minimal level. However, the short and long-term impact of this procedure on the local environmental bacterial communities and pathogen colonization/survival remains unknown. The present study monitored the natural biofilm community composition at various locations throughout one beef plant before and after the IS procedure as well as its impact on *S. enterica* colonization and survival within the mixed biofilms. Since the wastewater drains at beef processing plants collect all spray chill, cleaning, or other liquid runoff from the sanitization of processing equipment and the environment, we used floor drain samples as convenient representatives of the microorganisms present in the local environment.

Our results showed that the IS procedure altered the environmental microbial diversity even though it did not affect the biofilm-forming ability of microorganisms harvested from certain drain locations which remained consistently as strong (drain #3) or weak biofilm formers (drains #1 and #11) regardless of the sampling time points. However, a trend of stronger biofilm formation by the post-IS samples compared to the respective pre-IS samples was observed at seven drain locations (drains #4, #5, #6, #7, #8, #9, and #10), mostly located in the middle section of the plant. Among these, drain #9 at the hotbox area is the only location where the 1-week post-IS sample formed significantly weaker biofilm than its pre-IS sample. However, 4 weeks after the IS procedure the microorganisms from drain #9 formed significantly higher biofilm mass than its pre-IS and 1-week post-IS samples. Such observation was likely due to the IS treatment that eliminated most of the environmental microorganisms, but the highly tolerant species survived and thrived after their recovery and the removal of their competitors in the environment.

Available results from other research fields have shown pathogen decline but rapid recolonization after enhanced intervention or sanitization. For instance, in a hospital setting, plumbing replacement in response to an outbreak of Carbapenem-resistant *Enterobacteriaceae* (CRE) led to a decline in CRE acquisition incidence, however, environmental recolonization by CRE and patient CRE acquisitions recurred rapidly (Decraene et al., 2018). In our present study, when the *S. enterica* strain was introduced into the environmental multispecies community, *Salmonella* colonization within mixed biofilms exhibited no significant difference among the pre-, 1-week, and 4-week post-IS samples harvested from the same drain locations, suggesting that the IS procedure did not affect the plant environmental biofilms to recruit the *S. enterica* strain once it was reintroduced.

*S. enterica* survival in mixed biofilms after QAC treatment was overall positively correlated with the total mixed biofilm matrix. For instance, low *Salmonella* survival was measured in pre- and post-IS samples from drains #1 and #11 that both formed weak biofilms across the three sampling time points, and viable *Salmonella* was reduced to the non-detectable level in mixed biofilms by the 4-week post-IS samples from drains #2 and #12 that formed significantly lower biofilm matrix than the respective samples from the previous two sampling trips. Of the three samples from drain #9, no viable *Salmonella* cells were detected in the QAC-treated biofilm formed by the 1-week post-IS sample (weak biofilm former) whereas surviving *Salmonella* cells were observed in its pre- and 4-week post-IS biofilms (both strong biofilm formers). Thus, the pattern of *Salmonella* survival, in this case, was consistent with the biofilm-forming ability of the samples collected from this drain at different time points. Such a trend of a higher amount of surviving *S. enterica* cells in stronger biofilms formed by post-IS samples than in biofilms by the pre-IS counterparts was also observed within other sample sets in this experiment, though not statistically significant (Table 2).

Meat plants frequently harbor a wide variety of environmental microorganisms due to their daily processing operations. Recent studies (Belk et al., 2022; Palanisamy et al., 2023) indicated that the environmental microbial communities in different areas within a meat facility were differentiated by factors of room type/function, temperature, the activity taking place in the area, and the potential sources of microbes. However, in the meantime, a core microbiome was established in facility drains among which many organisms were capable of forming biofilms. This is consistent with our findings that *Pseudomonas* and *Microbacterium* were dominant in all drain samples across the three sampling time points, and *Streptococcus*, *Rhodococcus*, and *Paenibacillus* occupied considerable abundance in the facility before the IS procedure. Thus, the mixed biofilm development represents the main mechanism that allows the core microorganisms to persist in the environment. Interestingly, it was reported previously that *Pseudomonas* and *Microbacterium* were among the most prevalent taxa that are frequently present in food processing facilities regardless of the food commodity (Xu et al., 2023).

**Figure 4:**
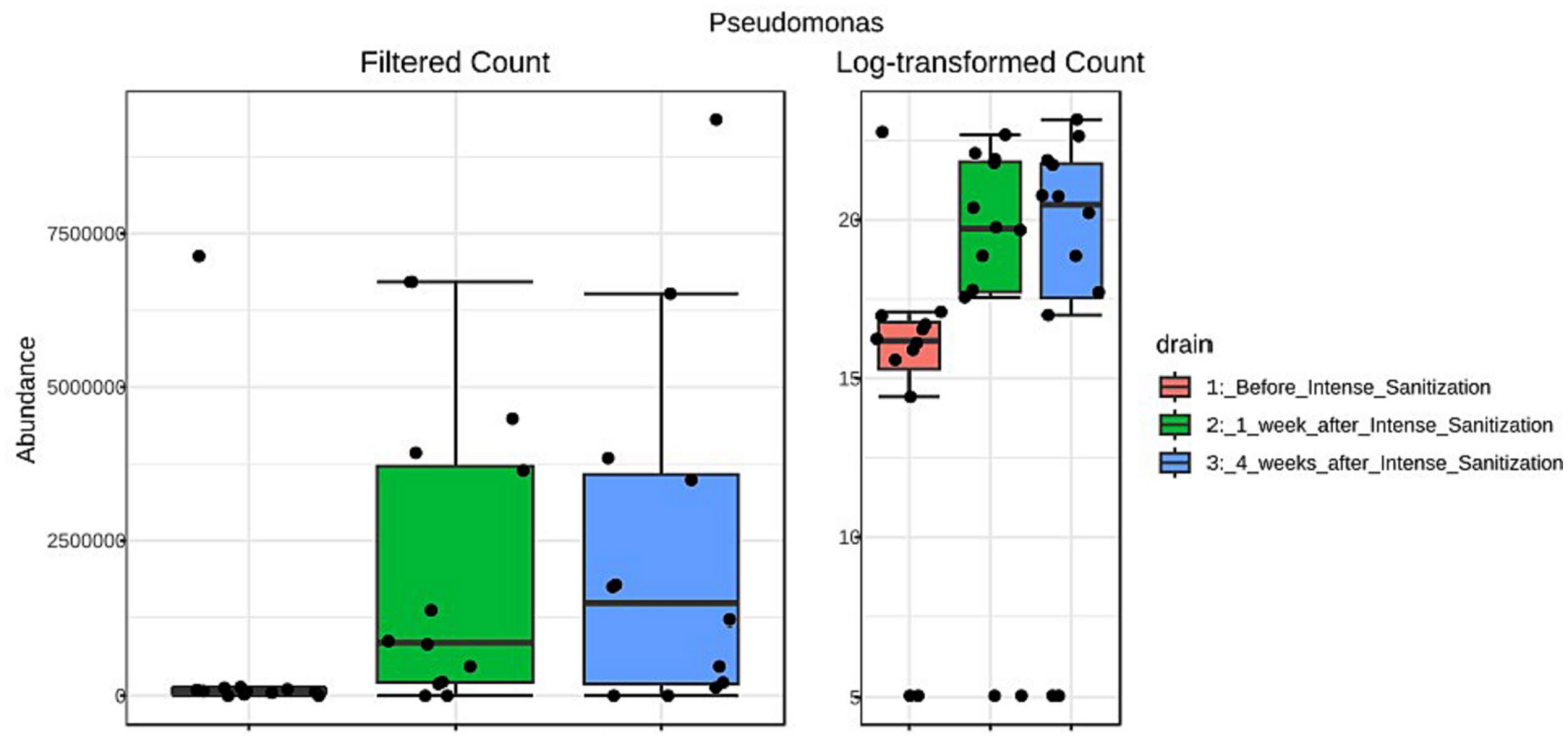
Linear discriminant analysis (LDA) effect size (LEfSe) analysis of *Pseudomonas* abundance reads in floor drain samples collected before and after IS procedure.

Furthermore, the unique local environmental conditions and external stress would also specially select certain organisms to thrive and dominate the local community. For instance, *Pseudomonas* was reported to be able to survive and outcompete companion organisms in areas of fabrication and processing/product holding areas where the temperature is low and nutrients are minimal due to their competitive advantage of strong tolerance against low temperature, high stress, and harsh growth conditions (Zhao et al., 2004; Belk et al., 2022). From a food facility after sanitization, *Pseudomonas* was also among the most abundant genera recovered that appeared to be strong biofilm producers at low temperatures (Langsrud et al., 2016). We obtained very similar observations in the present study. *Pseudomonas* abundance increased 1 week after IS and further increased substantially 4 weeks after the sanitization in drain samples collected from multiple locations (combo bin storage, cooler, fabrication floor, hot beef hall), many of which exhibited stronger biofilm-forming ability than their pre-IS counterparts and higher *Salmonella* survival in some of these post-IS mixed biofilms was observed. The LEfSe analysis further indicated that *Pseudomonas* was the only genus significantly (*p* = 0.027, LDA = 5.94) enriched after IS with the abundance of reads increasing from 657,200 (pre-IS) to 2,398,200 four weeks after IS (Figure 4). Interestingly, we recently observed that *Pseudomonas* was the most abundant genus in a beef plant with a *Salmonella* reoccurrence history (Wang et al., 2022). Other studies (Pang et al., 2017, 2020) further indicated that the presence of *Pseudomonas* in dual-species mixed biofilms could increase QAC tolerance of the companion *Salmonella* cells, which might lead to enhanced *Salmonella* prevalence in processing plants. Therefore, the potential correlation between *Salmonella* prevalence and the increased *Pseudomonas* presence in the environment after IS warrants further investigation.

The role of bacterial diversity in stabilizing environmental microbial communities and preventing the invasion of certain pathogenic species has been demonstrated (Kennedy et al., 2002; Blaser and Falkow, 2009; Blaser, 2016). Mahnert et al. (2019) showed that increasing cleaning and antimicrobial intervention could result in a loss of microbial diversity and a population shift from Gram-positive to Gram-negative bacteria. In the present study, drain samples collected 1 week post-IS had the least alpha diversity, which is an expected result of this treatment eliminating the majority of the environmental microorganisms and is also in agreement with the previous findings. Despite the decrease in the overall diversity, an abundance increase in certain species was observed, likely due to the inactivation of the weaker biofilm formers that were less tolerant of the procedure, and in the meantime, the strong biofilm formers survived the treatment and thrived within a less competitive environment.

Four weeks after the IS procedure, alpha diversity significantly increased (*p* < 0.05, Table 3; Figure 3) and reached the highest diversity level among the three sample groups. Meanwhile, beta diversity analysis using the PERMANOVA test indicated that the environmental microbial composition was significantly (*p* < 0.05) altered from the community prior to the IS procedure (Table 4). Such observation is not surprising since the meat processing plants represent a highly dynamic environment and the wide variety of bacterial species introduced by the incoming animals and the frequent personnel/processing activities on a daily basis could result in a shift in bacterial community structure and lead to a higher species diversity and altered community composition.

Previous studies suggested that the disruption and alteration of preexisting environmental microbial communities might lead to unintended consequences as a result of the elimination of the less tolerant species and the subsequent replacement by less desirable/invasive populations (Kotay et al., 2020). The previously described phenomena of pulse dynamics and disturbance in microbial ecology (Jentsch and White, 2019; Burton et al., 2020) may also shed light on a better understanding of our observation. The beef processing plant represents a built environment, and the natural microbial community has adapted over time within the specific ecological niche and developed tolerance to the daily common sanitization procedure, which was evident from our previous studies (Chitlapilly Dass et al., 2020; Palanisamy et al., 2023). A sudden perturbation such as the IS procedure (a “pulse disturbance”) will temporarily disturb the microbial community structure and lead to a temporary shift in the community composition (Supp and Morgan Ernest, 2014; Jentsch and White, 2019) which may have favored the retention of the species that demonstrate resilience to IS. Consequently, these abrupt changes in environmental conditions and disturbances influenced the microbial community structure, leading to the formation of stronger environmental mixed biofilms and enhanced protection for pathogens.

Meanwhile, the complexity and variability of the interactions within the mixed biofilms may increase with the species diversity as observed in the present study. This may lead to a more complex biofilm structure and a potentially more comprehensive tolerance mechanism resulting from the cooperation among the various species in the community as we previously demonstrated that higher species diversity and their ability to adhere, compete, and form mixed biofilms might impact pathogen tolerance in the mixture (Chitlapilly Dass et al., 2020). Thus, the recruitment of strong biofilm formers and the thriving of the tough survivors after a prolonged period of IS treatment could lead to an altered, overall higher diversified and stronger biofilm-forming community, which in turn might influence pathogen survival in the environment.

In summary, our study suggested that interventions unselectively removing/inactivating the majority of the microbes in the environment could alter the microbial community and cause unexpected effects as the resulting community structure shift may influence the overall ecologic and metabolic functions of the microbiome and promote colonization and overgrowth of undesired species. These changes may subsequently have an unintended impact on long-term pathogen prevalence and survival.

## Data availability statement

The authors declare that the data supporting the findings of this study are presented within the article. The fastq.qz files of the Illumina paired-end sequencing and metadata are originated from meat processing plants, and authors are obliged to maintain confidentiality, preventing the public deposition of these sequences. However, in case of a reasonable request, the authors are fully capable and willing to make these data available through file sharing. The data was generated under a non-disclosure agreement with meat processors, and we can share data with those willing to be bound by the same non-disclosure agreement.

## Author contributions

RW: Conceptualization, Data curation, Formal analysis, Funding acquisition, Investigation, Methodology, Supervision, Writing – original draft, Writing – review & editing. MG: Data curation, Investigation, Writing – review & editing. SC: Data curation, Formal analysis, Investigation, Software, Supervision, Writing – review & editing. VP: Data curation, Formal analysis, Investigation, Software, Writing – review & editing. JB: Conceptualization, Data curation, Formal analysis, Funding acquisition, Investigation, Methodology, Resources, Supervision, Writing – review & editing.

## Footnotes

^1^ https://data.qiime2.org/2022.8/common/silva-138-99-nb-classifier.qza

^2^ https://www.microbiomeanalyst.ca/

## Author disclaimer

The article was prepared by the employee of the U.S. Department of Agriculture as part of his/her official duties and therefore, is a work of the U.S. Government for which copyright is not available in the United States, as provided by Title 17 of the U.S. Code, Section 105. Accordingly, there is no domestic copyright to transfer. The fact that the publication in which the article appears is itself copyrighted does not affect the U.S. Government material, which may be reproduced freely by the public in the United States.

Publisher may secure copyright in the article to the extent authorized under the domestic laws of a foreign country, subject to a royalty-free, nonexclusive, irrevocable worldwide license to the United States in such copyrighted article to reproduce, prepare derivative works, distribute copies to the public and perform publicly and display publicly the article, and to permit others to do so.
